# Radiomic Features of Mesorectal Fat as Indicators of Response in Rectal Cancer Patients Undergoing Neoadjuvant Therapy

**DOI:** 10.3390/tomography11040044

**Published:** 2025-04-07

**Authors:** Francesca Treballi, Ginevra Danti, Sofia Boccioli, Sebastiano Paolucci, Simone Busoni, Linda Calistri, Vittorio Miele

**Affiliations:** 1 Department of Radiology, Careggi University Hospital, 50141 Florence, Italy; ginevra.danti@gmail.com (G.D.); sofia.boccioli@gmail.com (S.B.); linda.calistri@unifi.it (L.C.); vmiele@sirm.org (V.M.); 2 Department of Health Physics, Careggi University Hospital, 50141 Florence, Italy; sebastiano.paolucci@unifi.it (S.P.); busonis@aou-careggi.toscana.it (S.B.); 3 Department of Experimental and Clinical Biomedical Sciences, University of Florence, 50141 Florence, Italy

**Keywords:** rectal cancer, locally advanced rectal cancer, mesorectal fatty tissue, pathological complete response, tumor response, chemoradiotherapy, neoadjuvant, magnetic resonance imaging, radiomic, segmentation

## Abstract

Background: Rectal cancer represents a major cause of mortality in the United States. Management strategies are highly individualized, depending on patient-specific factors and tumor characteristics. The therapeutic landscape is rapidly evolving, with notable advancements in response rates to both radiotherapy and chemotherapy. For locally advanced rectal cancer (LARC, defined as up to T3–4 N+), the standard of care involves total mesorectal excision (TME) following neoadjuvant chemoradiotherapy (nCRT). Magnetic resonance imaging (MRI) has emerged as the gold standard for local tumor staging and is increasingly pivotal in post-treatment restaging. Aim: In our study, we proposed an MRI-based radiomic model to identify characteristic features of peritumoral mesorectal fat in two patient groups: good responders and poor responders to neoadjuvant therapy. The aim was to assess the potential presence of predictive factors for favorable or unfavorable responses to neoadjuvant chemoradiotherapy, thereby optimizing treatment management and improving personalized clinical decision-making. Methods: We conducted a retrospective analysis of adult patients with LARC who underwent pre- and post-nCRT MRI scans. Patients were classified as good responders (Group 0) or poor responders (Group 1) based on MRI findings, including tumor volume reduction, signal intensity changes on T2-weighted and diffusion-weighted imaging (DWI), and alterations in the circumferential resection margin (CRM) and extramural vascular invasion (EMVI) status. Classification criteria were based on the established literature to ensure consistency. Key clinical and imaging parameters, such as age, TNM stage, CRM involvement, and EMVI presence, were recorded. A radiomic model was developed using the LASSO algorithm for feature selection and regularization from 107 extracted radiomic features. Results: We included 44 patients (26 males and 18 females) who, following nCRT, were categorized into Group 0 (28 patients) and Group 1 (16 patients). The pre-treatment MRI analysis identified significant features (out of 107) for each sequence based on the Mann–Whitney test and *t*-test. The LASSO algorithm selected three features (shape_Sphericity, shape_Maximum2DDiameterSlice, and glcm_Imc2) for the construction of the radiomic logistic regression model, and ROC curves were subsequently generated for each model (AUC: 0.76). Conclusions: We developed an MRI-based radiomic model capable of differentiating and predicting between two groups of rectal cancer patients: responders and non-responders to neoadjuvant chemoradiotherapy (nCRT). This model has the potential to identify, at an early stage, lesions with a high likelihood of requiring surgery and those that could potentially be managed with medical treatment alone.

## 1. Introduction

Rectal cancer is a leading cause of cancer-related mortality in the United States, underscoring its significant impact on public health and the healthcare system. Its management is highly nuanced and individualized, with treatment strategies influenced by both patient-specific factors—such as overall health, age, and comorbidities—and tumor-specific characteristics, including size, location, and the extent of spread. These factors collectively guide a multidisciplinary team in formulating the most effective treatment plan [1,2,3].

The therapeutic landscape for rectal cancer is continually evolving, propelled by ongoing research and clinical advancements that have markedly improved patient outcomes [4]. Innovations in radiotherapy and chemotherapy have enhanced response rates, providing new hope for patients, particularly those with locally advanced rectal cancer (LARC). LARC is characterized by tumors T2 with regional lymph node involvement (N+) and T3–T4 with or without N+ for which a multimodal approach is standard practice to achieve optimal oncologic results [5,6].

Specifically, the established treatment protocol for LARC involves neoadjuvant chemoradiotherapy (nCRT) to reduce tumor size and improve surgical outcomes, followed by total mesorectal excision (TME)—a surgical technique designed to remove the rectum and its surrounding mesorectal tissue completely. This treatment sequence is pivotal for minimizing local recurrence and maximizing long-term survival [7,8,9,10]. Robotic surgery is an evolving technique that addresses some of the technical challenges of conventional laparoscopy, enhancing the precision and effectiveness of radical rectal cancer surgeries [11].

**Figure 1:**
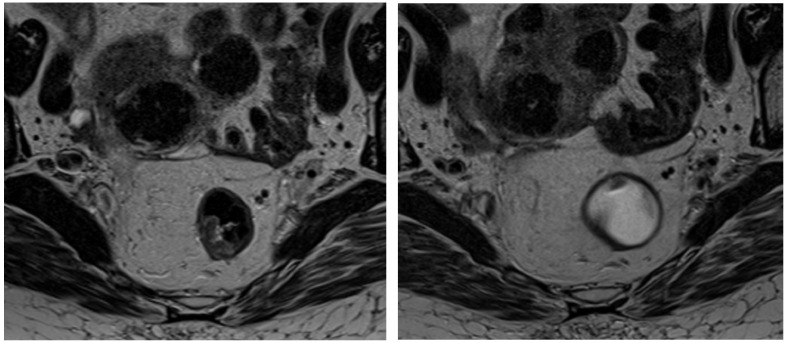
Staging MRI of rectal cancer before CRT (**left image**) and follow-up MRI after treatment (**right image**).

Magnetic resonance imaging (MRI) has become an indispensable tool in managing rectal cancer, particularly for local tumor staging before treatment [12,13,14,15,16]. Its superior imaging capabilities allow for detailed visualization of the tumor and adjacent structures, facilitating a precise assessment of tumor extent and tissue involvement. Moreover, MRI is increasingly utilized in the post-treatment setting for restaging and evaluating the response to neoadjuvant therapy, which is critical for guiding subsequent treatment decisions and optimizing patient outcomes (Figure 1) [17,18].

Mesorectal fat represents a crucial anatomical component in rectal cancer patients, serving both as the microenvironment for tumor invasion and as a key factor in risk stratification and treatment planning—especially for those undergoing neoadjuvant therapy.

In this context, radiomics has emerged as a promising technology that enables the quantitative extraction of features from diagnostic imaging modalities such as CT and MRI. These features, often imperceptible to the human eye, provide unique insights into the radiographic phenotype of the tumor and its microenvironment [19,20].

Prior studies have demonstrated its utility in distinguishing malignant from benign tissue in MR imaging, assessing cancer aggressiveness, and predicting responses to chemoradiotherapy (CRT) [21,22]. Thus, radiomics serves as a potential imaging biomarker for forecasting cancer progression and mortality.

Applying radiomics to mesorectal fat could yield valuable predictive information regarding the response to neoadjuvant therapy and overall clinical outcomes [22,23].

Our study aims to integrate radiomic analysis of mesorectal fat features with clinical risk stratification in rectal cancer patients, both before and after neoadjuvant therapy. The primary goal is to identify predictive features that can enhance the personalization of therapeutic strategies, ultimately optimizing clinical management and improving patient outcomes.

## 2. Materials and Methods

### 2.1. Study Population

We conducted a retrospective, single-center study on patients diagnosed with locally advanced rectal cancer (LARC) who underwent neoadjuvant radiochemotherapy. All patients were treated and monitored at the Radiology Department of Careggi University Hospital (Florence, Italy) between March 2020 and March 2024. Each patient received two MRI scans, spaced 10–12 weeks apart: the first for initial staging and the second for evaluating therapeutic response.

Neoadjuvant therapy consisted of radiotherapy (typically 45–50 Gy delivered in 25–28 fractions over 5 days per week) in combination with chemotherapy, most commonly using 5-Fluorouracil (5-FU) or Capecitabine. The clinical target volume (CTV) for radiotherapy included the primary rectal tumor, perirectal and internal iliac lymph nodes, mesorectum, pelvic sidewalls, and presacral space, with the superior boundary set at the sacral promontory. Following the second MRI, patients either proceeded to surgery or were managed non-surgically based on their individual needs and therapeutic response.

The inclusion criteria were as follows:

- A confirmed diagnosis of locally advanced rectal cancer.
- Completion of both pre- and post-neoadjuvant radiochemotherapy MRI evaluations performed at our center.

Exclusion criteria included as follows:

- Patients under 18 years of age.
- Missing either the pre- or post-treatment MRI scans.
- Poor-quality images on the T2-weighted axial sequence of the staging MRI.
- T4b staging at the time of diagnosis.

Patients were categorized into two groups based on their response to neoadjuvant therapy, as determined by follow-up MRI findings. Group 0 (good responders) included patients who demonstrated a significant reduction in tumor size and/or signal intensity changes suggestive of a favorable response to treatment. In contrast, Group 1 (poor responders) comprised patients with minimal or no tumor shrinkage and persistent high-signal intensity, indicating little to no response. The assessment of therapeutic response was conducted using standardized MRI criteria, which included changes in tumor volume, signal intensity on T2-weighted and diffusion-weighted imaging (DWI), and alterations in the circumferential resection margin (CRM) and extramural vascular invasion (EMVI) status. These MRI-based indicators were selected in accordance with established literature to ensure consistency and clinical relevance. For each patient, key clinical and imaging parameters were recorded, including age, TNM stage, CRM involvement, and EMVI presence. Tumor size was measured on axial T2-weighted images, and signal intensity changes were evaluated on T2-weighted and diffusion-weighted images.

### 2.2. Pathological Case Selection and Classification

Current guidelines recommend neoadjuvant therapy before total mesorectal excision (TME) for patients with LARC (T2 + N+ and T3–T4 ± N+). T3 tumors extend beyond the muscularis propria into the mesorectal tissues, while T4 tumors involve adjacent structures. Specifically, T4a tumors invade the peritoneal reflection, and T4b tumors extend into other organs or structures; T4b cases were excluded from this study. Nodal staging (N) was determined by the number of involved regional lymph nodes, including mesorectal and internal iliac nodes.

Patients were eligible regardless of the rectal segment affected (upper, middle, or lower), provided that there was no anal sphincter involvement. Demographic information, including age and sex, was obtained from clinical records.

### 2.3. Imaging Acquisition

MRI scans were performed using Siemens 1.5 T scanners (Aera and Magnetom Sola, SRN: 84081) with external coils for the lower abdomen. The imaging protocol, used for both baseline and follow-up examinations, included the following sequences:

- High-resolution T2-weighted imaging: Acquired in oblique axial, sagittal, and oblique coronal planes aligned with the rectal canal (small FOV: 180 mm; slice thickness ≤3 mm; 2D FSE without fat saturation; TR: 3800 ms; TE: 101 ms).
- Oblique axial diffusion-weighted imaging (DWI): Performed with b-values of 0, 500, and 1000 s/mm^2^ (small FOV: 320 mm) with ADC map reconstruction (EPI; TR: 6800 ms; TE: 56 ms).
- T1-weighted pelvic axial imaging: Used for evaluating iliac and paraaortic lymph nodes (large FOV: 310 mm; TR: 453 ms; TE: 7.8 ms).

Intravenous paramagnetic contrast was not routinely administered [24]. Patients were instructed to perform a small enema the day before imaging [25]. Additionally, 50 mL of endoluminal gel was administered during the scan to minimize mesorectal fat compression, thereby enhancing image quality.

### 2.4. Imaging Analysis

Two radiologists with over three years of experience in abdominal imaging independently evaluated the MRI scans, blinded to clinical and laboratory data. A senior radiologist with more than a decade of experience reviewed any cases of discrepancy. Image evaluation was conducted in two phases: first, by comparing pre- and post-neoadjuvant therapy examinations for each patient; second, by categorizing patients into two groups based on therapeutic response—Group 0 (complete or good responders) and Group 1 (incomplete or non-responders).

### 2.5. Radiomic Workflow

**Figure 2:**
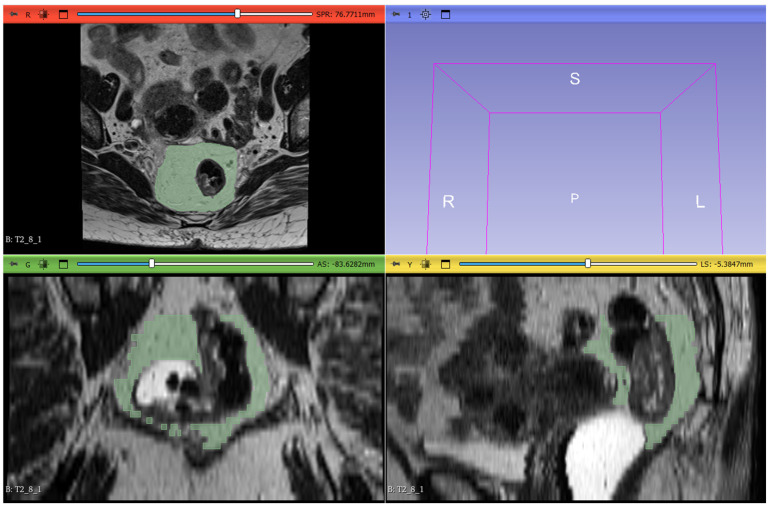
The image-processing software 3DSlicer was used to manually delineate ROIs along the lesion margins on all slices containing the tumor in the MRI T2w sequences (red, green, and yellow squares), as illustrated in the figure. The volumetric 3D reconstruction of the tumor is displayed in the upper—right quadrant (light blue square).

The radiomic workflow included lesion segmentation, feature extraction, and feature selection. T2-weighted images from the staging MRI were imported into 3D Slicer software (version 5.6.1) for segmentation. Radiologists manually delineated the mesorectal fat on all slices, defining the region of interest (ROI). The proximal boundary was defined at the point where the anterior peritoneal reflection attaches to the rectal wall (visible as a V-shaped structure on the axial plane), and the distal boundary was set at the last visible portion of mesorectal fat above the intersphincteric plane. The segmentation excluded the rectum, the tumor, any tumor deposits within the mesorectal fat, pathological lymph nodes, and mesorectal vessels invaded by the disease (Figure 2).

Using the PyRadiomics module (version 3.1.0), quantitative radiomic features were extracted from each ROI and categorized into three main classes:

- Shape-based features: Describe the three-dimensional size and geometry of the ROI.
- First-order statistics: Represent the voxel intensity distribution within the ROI.
- Second-order statistics: Capture the spatial correlation of intensity values within the ROI.

Statistical analysis was subsequently performed to identify significant features for model development.

### 2.6. Statistical Analysis

Statistical analyses were performed using R software (version 4.1.1; https://www.r-project.org, accessed on 21 June 2023). Depending on the homogeneity of variances (assessed by Levene’s test) and normality of data distribution (assessed by Shapiro’s test), either the *T-*test or the non-parametric Mann–Whitney test was applied to identify features with statistically significant differences between the two groups (*p* < 0.05 considered significant). A logistic regression model based on radiomic features was constructed using the least absolute shrinkage and selection operator (LASSO) method for simultaneous feature regularization and selection. Receiver Operating Characteristic (ROC) curves and Area Under the Curve (AUC) values, along with 95% confidence intervals (CI), were generated for the T2-weighted sequences from the pre-neoadjuvant MRI examinations.

### 2.7. Compliance with Ethical Standards

The authors declare no conflicts of interest. All patients signed an informed consent form. The study was approved by the Biomedical Research Ethics Committee of our institution in accordance with the criteria of the Declaration of Helsinki on Ethical Principles and GoodClinical Practice.

## 3. Results

A total of 68 patients with a confirmed diagnosis of rectal cancer were initially selected for this study. These patients underwent MRI at our center between March 2020 and March 2024, with a median follow-up duration of 10–12 weeks. Of these, 24 patients were excluded due to one or more of the following reasons: insufficient image quality for segmentation, one of the two MRI scans being performed on a different scanner, or the second MRI not being completed, thus precluding the evaluation of neoadjuvant therapy response.

Consequently, the final cohort consisted of 44 patients (26 males and 18 females) with a mean age at diagnosis of 69 ± 10.3 years (refer to Table 1 for demographic data). Based on the TNM staging system at baseline, 3 patients (6.8%) were classified as stage T2, 33 patients (75%) as stage T3, and 8 patients (18.2%) as stage T4.

Patients were subsequently stratified into two groups according to their response to neoadjuvant therapy: Group 0 included patients with a complete or good response (28 patients), while Group 1 comprised patients with an incomplete or poor response (16 patients). Within Group 0, baseline staging revealed 3 patients in stage T2, 23 in stage T3, and 4 in stage T4. In Group 1, no patients were classified as stage T2, 10 were stage T3, and 6 were stage T4 (Table 2).

The behavior of 107 extracted radiomic features was then analyzed to identify those that differed significantly between the two groups. Initially, the Shapiro–Wilk and Levene tests were applied to assess the normality and homogeneity of variances, respectively. Depending on these results, either a *t*-test or a Mann–Whitney test was used to evaluate statistical significance.

Focusing on baseline MRI data (pre-nCRT), the analysis identified two features as significant using the Mann–Whitney test: glcm_Correlation (*p* = 0.0349) and glcm_Imc2 (*p* = 0.035), both second-order features derived from the Gray Level Co-occurrence Matrix (GLCM). However, neither feature remained statistically significant after applying Bonferroni correction for multiple testing (adjusted significance threshold = 0.000467).

**Figure 3:**
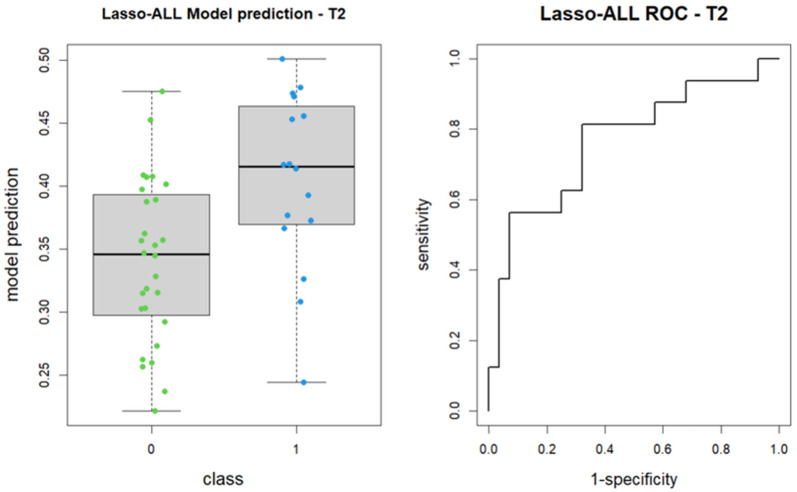
Boxplot of performance in prediction models (**left**) and ROC curves (with AUC) associated to prediction models (**right**); prediction models were calculated with LASSO regression method on baseline images (T2w). Each colored dot represents the probability of membership in group 1 for each patient.

Subsequently, the LASSO algorithm was employed to construct a logistic regression model for feature regularization and selection from the 107 features. The algorithm identified shape_Maximum2DDiameterSlice and glcm_Imc2 as the most predictive features for patient group classification, although the former did not reach statistical significance. The model achieved an area under the receiver operating characteristic curve (AUC) of 0.766, with a 95% confidence interval ranging from 0.61 to 0.922 (DeLong method) (Figure 3).

A segmentation review was then performed on the same patients using the T2-weighted baseline MRI sequences, following the methodology of Jayaprakasam et al. [26]. Repeating the statistical analysis on these revised segmentations yielded similar results, with glcm_Correlation (*p* = 0.0303) and glcm_Imc2 (*p* = 0.0146) emerging as significant features. Although these features demonstrated improved significance compared to the initial segmentation, they still did not meet the Bonferroni-corrected threshold.

**Figure 4:**
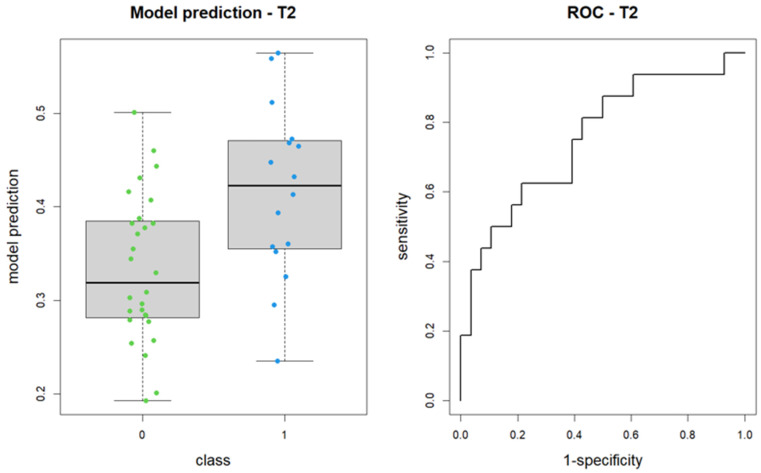
Boxplot of performance in prediction models (**left**) and ROC curves (with AUC) associated to prediction models (**right**) repeated after segmentation review; prediction models were calculated again with LASSO regression method on baseline images (T2w). Each colored dot represents the probability of membership in group 1 for each patient.

Upon reapplying the LASSO method for logistic regression model construction on the revised segmentation data, the selected features included shape_Sphericity, shape_Maximum2DDiameterSlice (both non-significant), and glcm_Imc2. The revised model produced an AUC of 0.76, with a 95% confidence interval of 0.60 to 0.91 (DeLong method) (Figure 4).

## 4. Discussion

In this study, an MRI-based radiomic model was developed to differentiate and predict responders and non-responders among 44 patients with locally advanced rectal cancer (LARC) who underwent neoadjuvant chemoradiotherapy (nCRT). Given the pivotal role of neoadjuvant therapy response in guiding subsequent treatment strategies, our approach leverages radiomic features extracted specifically from the peritumoral mesorectal fat—a region increasingly recognized for its critical influence on tumor behavior and treatment outcomes.

The ability to predict therapeutic response in LARC is crucial as it can help tailor treatment strategies, minimizing unnecessary surgical interventions while maximizing patient outcomes. Following neoadjuvant chemoradiotherapy (nCRT), approximately 60% of patients exhibit favorable downstaging, with around 10% achieving a pathological complete response (pCR). These outcomes are of particular significance in advancing non-surgical management strategies, such as the watch-and-wait approach, which has gained traction in clinical settings. Key histopathological factors, including fibrosis, tumor necrosis, mucin production, and residual tumor presence, have been identified as significant contributors to these outcomes [27]. Notably, mucinous rectal carcinomas, which are often resistant to nCRT due to their distinct histopathological properties, were categorized as Group 1 in our study [1,28]. The unique role of peritumoral mesorectal fat as a microenvironmental hub for tumor interactions motivated its inclusion as a primary target in our radiomic analysis. Given the growing interest in tumor microenvironment characterization, understanding the mesorectal fat’s textural attributes could provide deeper insights into treatment response variability.

Our model analyzed both pre- and post-nCRT MRI data, focusing on radiomic features extracted from the mesorectal fat. The application of these features enabled the categorization of patients into two macro-groups: good and poor responders. Employing the Least Absolute Shrinkage and Selection Operator (LASSO) method, a predictive logistic regression model was developed that achieved an area under the curve (AUC) of 0.76. Among the features identified, glcm_Correlation and glcm_Imc2 were found to be clinically relevant, emphazising the significance of texture-related metrics in capturing the structural and informational complexity of peritumoral mesorectal fat.

- glcm_Correlation reflects the degree of linear dependency in pixel intensity relationships within the gray level co-occurrence matrix (GLCM). High values suggest strong linear correlations and regular patterns, thereby effectively distinguishing between normal and pathological tissues.
- glcm_Imc2 quantifies the informational correlation between pixel pairs through the joint entropy of the GLCM. Higher values indicate ordered and predictable textures, whereas lower values denote complex, irregular structures typically associated with pathological changes.

Interestingly, shape-related features did not contribute significantly to patient group differentiation, thereby emphasizing the predictive power of texture-based metrics derived from peritumoral mesorectal fat. This finding is consistent with the results of earlier research by Jayaprakasam et al., Li et al., and Yardimci et al., which demonstrated the utility of axial T2-weighted images in developing predictive models focused on treatment response and pCR in rectal cancer patients [26,29,30]. The lack of significance of shape-based features suggests that tumor morphology alone may be insufficient to capture the full extent of treatment response heterogeneity, reinforcing the necessity of advanced radiomic approaches.

By prioritizing the analysis of peritumoral mesorectal fat, our study underscores the potential of radiomics to refine early therapeutic decision-making. This approach may enable clinicians to more accurately identify patients who would benefit from surgical intervention as opposed to those suitable for a watch-and-wait strategy.

Nevertheless, the present study is not without its limitations, including a small patient sample, a monocentric and retrospective design, and reliance on a single MRI scanner. While these constraints ensured data uniformity, they underscore the need for further research. The expansion of the sample size, the incorporation of data from multiple centers, the exploration of diverse nCRT regimens, and the integration of additional clinical data could enhance the model’s generalizability. The application of deep learning techniques, such as convolutional neural networks (CNNs), holds promise for further refining feature extraction and enhancing predictive accuracy. Such advancements have the potential to pave the way for a comprehensive radiomic nomogram that supports personalized and precise treatment strategies for rectal cancer patients. Future research should also explore the potential of radiomic–genomic correlations, shedding light on the molecular mechanisms underlying differential treatment responses and paving the way for truly individualized oncologic care.

## 5. Conclusions

In summary, the MRI-based radiomic model developed in this study demonstrates significant potential for predicting therapeutic responses in patients with locally advanced rectal cancer (LARC). By leveraging radiomic features extracted from the peritumoral mesorectal fat—specifically, glcm_Correlation and glcm_Imc2—this approach captures the microenvironmental dynamics that are critical to tumor behavior and treatment outcomes. These features facilitate early differentiation between lesions likely to require surgical intervention and those that may be managed effectively with medical therapy alone.

This methodology holds promise for reducing the need for surgery in selected patients and optimizing clinical management from the time of diagnosis. Moreover, it aligns with our goal of integrating radiomic analysis of mesorectal fat features with clinical risk stratification to enhance the personalization of therapeutic strategies, ultimately leading to more tailored treatment pathways and improved patient outcomes.

Looking ahead, efforts will focus on refining and expanding the model to ensure its broader accessibility and seamless integration into routine clinical practice. Future developments will aim to create a user-friendly, adaptable tool capable of supporting real-time decision-making by clinicians, thereby reinforcing the role of radiomic features in advancing precision medicine for rectal cancer care.

## Figures and Tables

**Table 1:** Demographic characteristics of the population at the baseline.

	N = 44
Males	26
Females	18
Median age at diagnosis	69
T2 *	3
T3 *	33
T4 *	8
CRM positive	20
EMVI positive	10

* According to TNM criteria.

**Table 2:** Baseline staging distribution by group.

	N = 44
Good responders (group 0)	28
T2	3
T3	23
T4	2
Poor responders (group 1)	16
T2	0
T3	10
T4	6

## Data Availability

Data are contained within the article.
